# Chinese Version of the mHealth App Usability Questionnaire: Cross-Cultural Adaptation and Validation

**DOI:** 10.3389/fpsyg.2022.813309

**Published:** 2022-02-02

**Authors:** Shuqing Zhao, Yingjuan Cao, Heng Cao, Kao Liu, Xiaoyan Lv, Jinxin Zhang, Yuxin Li, Patricia M. Davidson

**Affiliations:** ^1^Department of Nursing, The Second Hospital, Cheeloo College of Medicine, Shandong University, Jinan, China; ^2^Department of Nursing, Qilu Hospital, Cheeloo College of Medicine, Shandong University, Jinan, China; ^3^School of Nursing and Rehabilitation, Shandong University, Jinan, China; ^4^Nursing Theory and Practice Innovation Research Center, Shandong University, Jinan, China; ^5^Department of Nursing, Shandong Provincial Hospital Affiliated to Shandong First Medical University, Jinan, China; ^6^The Vice-Chancellor’s Unit, University of Wollongong, Wollongong, NSW, Australia

**Keywords:** mHealth apps, content validity index, cross-cultural adaptation, questionnaire translation, usability testing tools

## Abstract

**Background:**

Mobile health (mHealth) apps have shown the advantages of improving medication compliance, saving time required for diagnosis and treatment, reducing medical expenses, etc. The World Health Organization (WHO) has recommended that mHealth apps should be evaluated prior to their implementation to ensure their accuracy in data analysis.

**Objective:**

This study aimed to translate the patient version of the interactive mHealth app usability questionnaire (MAUQ) into Chinese, and to conduct cross-cultural adaptation and reliability and validity tests.

**Methods:**

The Brislin’s translation model was used in this study. The cross-cultural adaptation was performed according to experts’ comments and the results of prediction test. The convenience sampling method was utilized to investigate 346 patients who used the “Good Doctor” (“Good Doctor” is the most popular mHealth app in China), and the reliability and validity of the questionnaire were evaluated as well.

**Results:**

After translation and cross-cultural adaptation, there were a total of 21 items and 3 dimensions: usability and satisfaction (8 items), system information arrangement (6 items), and efficiency (7 items). The content validity index was determined to be 0.952, indicating that the 21 items used to evaluate the usability of the Chinese version of the MAUQ were well correlated. The Cronbach’s α coefficient of the total questionnaire was 0.912, which revealed that the questionnaire had a high internal consistency. The values of test-retest reliability and split-half reliability of the Chinese version of the MAUQ were 0.869 and 0.701, respectively, representing that the questionnaire had a good stability.

**Conclusion:**

The translated questionnaire has good reliability and validity in the context of Chinese culture, and it could be used as a usability testing tool for the patient version of interactive mHealth apps.

## Introduction

The Global Observatory for eHealth of the World Health Organization (WHO) defined mHealth as “medical and public health practice supported by mobile devices, such as mobile phones, patient monitoring devices, personal digital assistants, and other wireless devices.” A large number of investigations and studies on mHealth apps have shown that mHealth apps can improve medication compliance, save time required for diagnosis and treatment, and reduce medical expenses (Seto et al., 2012; Fairman et al., 2013; Parmanto et al., 2013; Pfammatter et al., 2016). With the aging of the population, demand for healthcare services has markedly increased, imposing a huge pressure on medical institutions (Association of American Medical Colleges [AAMC], 2021). According to a previous research, the Internet-based treatment could noticeably reduce the number of outpatient visits (National Health Service [NHS], 2019). It could also decrease the burden on hospital resources, medical staff’s workload, and medical expenses (Shah et al., 2021). Besides, mHealth apps have shown a potential to greatly improve healthcare systems.

China has a large population and relatively few medical resources. Faced with the fact that the development of mHealth apps can relieve the tension of medical institutions, the government has put forward the development of the Internet-based healthcare services. The coronavirus disease 2019 (Covid-19) pandemic has caused the urgent need to redesign public health systems from reactive to proactive and to develop innovations that may provide real-time information for making proactive decisions. Owing to the limited healthcare resources, several national healthcare providers are turning to digital healthcare solutions, such as virtual ward, remote patient monitoring, and telemedicine, to minimize the risk of Covid-19, in which mHealth apps have shown a noticeable efficiency (Mann et al., 2020; Schinkothe et al., 2020).

According to the published statistics, more than 325,000 mHealth apps have been universally developed in recent years (Maramba et al., 2019). However, nearly half of the million mHealth programs that have been developed are not extensively utilized. Because there was no effective usability evaluation of mHealth apps, clinicians, scholars, and patients are skeptical about the reliability of mHealth programs (Vera et al., 2019). These limitations have negatively influenced the applicability of mHealth apps. Hence, the WHO has recommended that mHealth apps should be evaluated prior to their actual use in order to ensure the accuracy in data analysis (World Health Organization [WHO], 2020). At the initial stage of the use of a mHealth app, usability assessment is the key, which can remarkably motivate individuals to use a mHealth app more easily and efficiently.

The definition of usability by the International Organization for Standardization (IOS) :the extent to which a system, product or service can be used by specified users to achieve specified goals with effectiveness, efficiency and satisfaction in a specified circumstance (International Organization for Standardization [ISO], 2018). To evaluate the usability of apps, qualitative methods are used previously, such as interviews (Stinson et al., 2006), cognitive walkthrough (Van Velsen et al., 2018) and heuristic evaluation (Walsh et al., 2017), etc. However, these methods are mainly for tracking usability problems. They do not calculate the absolute score of a system’s usability, which can be achieved via usability tests instead. A usability test is a clear indicator to show whether the usability of an app is sufficient or insufficient (Broekhuis et al., 2021). Ideally, usability test should be conducted at each step of the program development, which includes an iterative cycle of system design and validation (Ivory and Marti, 2001). Testing the usability of an app is most frequently done by means of usability questionnaires (Peute et al., 2008).

The usability Questionnaire is the most common tool for usability assessment, as it is user-friendly and the data analysis is intuitive (Sevilla-Gonzalez et al., 2020). There are currently many usability questionnaires that are validated and reliable, such as Questionnaire for User Interface Satisfaction (Chin et al., 1988),the Post-Study System Usability Questionnaire (PSSUQ) (Lewis, 2002), Perceived Usefulness and Ease of Use (Davis, 1989), System Usability Scale (SUS) (Brooke, 1996), and Usefulness, Satisfaction, and Ease of Use Questionnaire (Lund, 2001). These questionnaires have been used widely in lots of mHealth app usability studies. However, none of them was specifically designed for evaluating the usability of mHealth apps (Zhou et al., 2017). Despite the large number of mHealth apps released to the public, there is still a lack of targeted evaluation tools. Therefore, Zhou et al. (2019) developed and validated a new mHealth app usability questionnaire (MAUQ), which is a targeted reliable usability testing tool in the mHealth domain. The MAUQ, based on the heterogeneity of definitions and methods, is used to evaluate the usability of mHealth apps. Three usability-based dimensions that were consistent with the definition were explored: Ease of use and satisfaction (corresponding to satisfaction of usability); System information arrangement (corresponding to the efficiency of availability); and practicability (corresponding to the availability).

MHealth apps can be divided into two different versions for patients and healthcare providers depending on the target audience. According to the interactive status of mHealth apps, they can be divided into interactive mHealth apps and standalone mHealth apps. Zhou et al. (2019) developed the MAUQ based on a number of existing questionnaires used in previous mobile app usability studies. Then, they utilized MAUQ, SUS, and PSSUQ to investigate the usability of two mHealth apps: an interactive mHealth app and a standalone mHealth app. Four versions of the MAUQ were developed in association with the type of app (interactive or standalone) and target user of the app (patient or provider). A website was created to make it convenient for developers of mHealth apps to use the MAUQ to assess the usability of their mHealth apps (PITT Usability Questionnaire, 2021).

MHealth app usability questionnaire has been widely utilized in the United States (Sengupta et al., 2020; Sood et al., 2020; Cummins et al., 2021), Sweden (Anderberg et al., 2019), Australia (Menon et al., 2019), Spain (Soriano et al., 2020), South Africa (Gous et al., 2020), and other countries. It is mainly used to study the usability of digital healthcare systems. Its standalone patient version has been translated into the Malaysian version, and it has shown an acceptable practicability (Mustafa et al., 2021). However, China has not yet introduced a Chinese version of the MAUQ, the present study aimed to translate and verify the patient version of the interactive mHealth app usability questionnaire (MAUQ) into Chinese, and to provide a usability testing tool for developers of mHealth apps in China.

## Materials and Methods

### Overview

Zhou et al. (2019) developed the MAUQ in 2019. It contains a total of 21 items and 3 dimensions: usability and satisfaction (8 items), system information arrangement (6 items), and efficiency (7 items). Cronbach’s alpha coefficients of the three dimensions of the original questionnaire were 0.895, 0.829, and 0.900, respectively, indicating a strong internal consistency of the MAUQ. The 7-point Likert scoring system was adopted as follows: 1 (extremely strongly agree), 2 (strongly agree), 3 (agree), 4 (neutral), 5 (disagree), 6 (strongly disagree), and 7 (extremely strongly disagree). The questionnaire score is the total score of each item divided by the number of items. The closer the mean value is to 1, the higher the app’s usability will be.

### Questionnaire Translation

It is important to contact a questionnaire’s developer(s) to obtain the permission. The author of the original questionnaire has been contacted and permission for translation and use has been obtained. The Brislin’s translation model (Brislin, 1976) was adopted as follows: Step 1. Forward translation (from English into Chinese): The questionnaire was translated by two translators with high English proficiency, and then, the research group and the two translators could jointly form the Chinese version of the MAUQ (A); Step 2. Back translation: Two translators who had not contacted the original questionnaire translated the Chinese version of the MAUQ (A) into English version, and the research group finalized the English version. After that, the research group and all the translators compared the translated version (From Chinese into English) with the original questionnaire, and the Chinese version of MAUQ (B) could be provided after discussing and performing the required amendments.

### Cross-Cultural Adaptation

Literal translation is not sufficient to produce an equivalent questionnaire, and cultural sensitivities or cultural influences may not cause problems, while they may lead to a misunderstanding of the question being asked, indicating the necessity of conducting cross-cultural adaptation (Epstein et al., 2015; Mohamad Marzuki et al., 2018). Therefore, we conducted cross-cultural adaptation according to experts’ comments and prediction test.

#### Experts’ Comments

Seven experts familiar with the field of mHealth were invited: two associate professors of medicine, one senior engineer of software design, one doctoral assistant researcher, one associate professor of nursing, and two master’s degree nurses. Two researchers of the group took back the expert opinions on site. According to their theoretical knowledge and practical experience, each expert evaluates the accuracy of translation, content comprehension, language expression habits and consistency on cultural background of each item one by one. For the suggestions that are not easy to understand, the researcher had an in-depth discussion with the experts and made records in detail. Finally, the group summarized all the opinions from experts.

#### Prediction Test

A preliminary survey was conducted on 30 patients who used the mHealth app of “Good Doctor” (“Good Doctor” is the most popular mHealth app in China, and this app showed a high utilization rate), using convenience sampling method, among outpatients of a third-class hospital, aiming to figure out respondents’ understanding of the questionnaire and their feelings on items of the questionnaire. The researcher explained the purpose of the survey to the respondents, made an investigation using the translated questionnaire, and asked the following questions: (1). Do you understand the content of this item? Or whether there is any ambiguity? (2). Do you know how to answer? If you don’t, what might be the difficulty? (3). Does it conform to Chinese language expression habits, if not, in which way you think it should be expressed? After the investigation and communication with the respondents, the items in the questionnaire that can be confusing and difficult to understand or answer were marked. The questions and suggestions raised by the respondents were recorded.

The Chinese version of MAUQ (B) was revised to form the final Chinese version of the MAUQ based on experts’ suggestions and respondents’ feedback.

### Research Objects and Research Tools

This cross-sectional, descriptive study was conducted in four large hospitals of Jinan, Shandong Province, China, from October 2020 to February 2021. The convenience sampling method was adopted to select users of the patient version of the “Good Doctor” mHealth app as research objects. Inclusion criteria were as follows: owning a smart phone and being proficient in mobile phone apps; users who aged ≥ 18, ≤ 65 years old, with good verbal and written communication skills; users who have used this app for diagnosis/treatment twice or more in the past month (Sevilla-Gonzalez et al., 2020). Exclusion criteria were as follows: language barriers or communication difficulties; being concomitant with psychiatric or neurological disorders. All participants gave informed consent and voluntarily participated in the study.

After obtaining the study approval from relevant departments, the research team members, who had received unified training, conducted the investigation. The research tools included users’ general information, involving users’ age, gender, place of residence, marital status, educational level, annual income, and the frequency of using “Good Doctor” mHealth app in the last 2 months. The purpose and significance of the questionnaire, as well as the confidentiality principle were explained to subjects before commencing the survey.

Two well-trained and eligible research investigators collected data. The collected questionnaires are numbered uniformly, and the data was entered by two people, excel used for data entry and SPSS 21 used for statistical analysis, and AMOS 23 statistical software used for confirmatory factor analysis to verify the appropriateness and stability of the construct validity of the scale.

### Verification of the Questionnaire

#### Item Analysis

Examine the central tendency of the answers to an item, if there is a choice in the answer set for an item and the percentage is more than 80%, indicating that the discrimination ability of the item is weak, and should be deleted. The total score of the questionnaire was arranged in a logical order. The *t*-test was used to examine the mean difference, and items with no significant difference between high-score and low-score groups were deleted. Using the Pearson’s correlation coefficient method, and according to the item score and the total score of the questionnaire, items with a very poor correlation with the total score of the questionnaire were deleted (*r* < 0.30).

#### Reliability and Validity Testing of the Questionnaire

Seven experts in mHealth were invited to evaluate the relevance of the questionnaire contents. The 4-point Likert scoring system was used to evaluate the correlation between each item and the questionnaire topic [1 (no correlation), 2 (poor correlation), 3 (strong correlation), and 4 (extremely strong correlation)]. The content validity index (CVI) of all items and the CVI of the total score of the questionnaire were calculated. Principal component analysis and maximum variance orthogonal rotation were utilized for exploratory factor analysis (EFA). Items with a load factor of < 0.40 were deleted. The correlation matrix between each dimension and the total score of the questionnaire was tested and the internal correlation analysis was carried out. Confirmatory factor analysis (CFA) was undertaken to verify whether the structure of the questionnaire was consistent with the theoretical structure of the original questionnaire. The reliability of the questionnaire was evaluated by the Cronbach’s α coefficient, test-retest reliability, and split-half reliability.

This study was approved by the Ethics Committee of Qilu Hospital of Shandong University (Jinan, China; Approval No. KYLL-2020-460).

## Results

### Cross-Cultural Adaptation

According to the experts’ comments and respondents’ feedback, related to content understanding, language expression habits, and cultural background of the questionnaire, and the results of prediction test, the research group modified the contents as follows: (1). The term “various social environments” in item 5 was not consistent with the language expression habits in China. Therefore, item 5 “I feel comfortable using this app in various social environments” was changed to “I feel comfortable using this app in various public places” (2). Item 6 “The time required to use the app is appropriate for me.” was changed to “Using this app will not take up much of my time” (3). The 12th item was not easy to understand after literal translation, thus, the item 12 “navigation is consistent when switching pages” was changed to “the way and process of switching pages are consistent”.

### General Information of the Subjects

A total of 444 electronic questionnaires were forwarded to users of the patient version of the “Good Doctor” mHealth app, and 346 questionnaires were effectively received with an effective recovery rate of 77.920%. Analysis of the results showed that, there were 186 and 160 male and female cases, accounting for 53.757% and 46.243% of the total sample size, respectively. In terms of age, the majority of users aged between 29 and 48 years old. The dominancy of bachelor’s degree was found among users. Besides, users mainly lived in urban areas. The proportion of “in-service” personnel was the highest. Table 1 summarizes further details.

**TABLE 1:** Subjects’ demographic characteristics (*n* = 346).

Characteristic		Frequency	Percentage (%)	Accumulated percentage (%)
Gender	Man	186	53.757	53.757
	Woman	160	46.243	100
Age (years old)	18–28	31	8.960	8.960
	29–38	115	33.237	42.197
	39–48	110	31.792	73.988
	49–65	90	26.012	100
Standard of culture	Junior high school degree or above	86	24.855	24.855
	High school degree	64	18.497	43.353
	Junior college	81	23.410	66.763
	Undergraduate degree	98	28.324	95.087
	Master degree or above	17	4.913	100
Place of residence	City	245	70.809	70.809
	Suburb	19	5.491	76.301
	Village	82	23.699	100
Profession	Student	4	1.156	1.156
	In-service staff	178	51.445	52.601
	Retirement	30	8.671	61.272
	Liberal professions	77	22.254	83.526
	Else	57	16.474	100
Household Income (CNY/Month)	<5000	161	46.532	46.532
	5000–10000¥	116	33.526	80.058
	10000–20000¥	40	11.561	91.618
	>20000¥	29	8.382	100
The frequency the utilizing medical apps in the last month	2 –3	47	13.584	13.584
	3–6	93	26.879	40.462
	6–12	72	20.809	61.272
	≥ 10	134	38.728	100
Total		346	100	100

### Item Analysis

Table 2 shows the results of the standalone-samples *t*-test, comparing differences in each item between high-score and low-score groups. Items with no statistically significant difference between high-score and low-score groups were deleted (Jones et al., 2001), and the results showed that significant differences existed in each item between high-score and low-score groups, thus, no item was deleted.

**TABLE 2:** Item analysis (standalone-samples *t*-test).

The total score was divided into high-score and low-score groups		*N*	Mean value	Standard deviation	The standard error of the mean	*t*	Sig.
A1. The app is easy to use	1.00	95.000	4.737	1.489	0.153	−9.980	0.000
	2.00	95.000	6.453	0.769	0.079		
A2. It was easy for me to learn how to use the app	1.00	95.000	4.621	1.474	0.151	−11.944	0.000
	2.00	95.000	6.600	0.659	0.068		
I like the interface of the app	1.00	95.000	4.632	1.481	0.152	−11.109	0.000
	2.00	95.000	6.526	0.756	0.078		
A4. I need that the information in this app is very organized and I	1.00	95.000	4.484	1.436	0.147	−12.126	0.000
can easily find the information	2.00	95.000	6.495	0.742	0.076		
A5. I feel comfortable using the app in all kinds of public places	1.00	95.000	4.568	1.389	0.142	−10.903	0.000
	2.00	95.000	6.368	0.813	0.083		
A6. Using this app doesn’t take much of my time	1.00	95.000	4.558	1.493	0.153	−10.949	0.000
	2.00	95.000	6.421	0.723	0.074		
A7. I will use the app again	1.00	95.000	4.853	1.845	0.189	−7.872	0.000
	2.00	95.000	6.432	0.647	0.066		
A8. Overall, I’m happy with the app	1.00	95.000	4.526	1.508	0.155	−10.336	0.000
	2.00	95.000	6.347	0.822	0.084		
B1. Whenever I have a wrong operation while using the app, I can	1.00	95.000	4.737	1.362	0.140	−10.085	0.000
easily and quickly correct it	2.00	95.000	6.379	0.814	0.084		
B2. The mHealth app offers healthcare in a way that is easy for	1.00	95.000	4.947	1.887	0.194	−6.924	0.000
users to accept	2.00	95.000	6.389	0.748	0.077		
B3. The app gives me enough feedback and information to let me	1.00	95.000	4.716	1.506	0.155	−9.900	0.000
know where my steps are going	2.00	95.000	6.453	0.809	0.083		
B4. The way that B4 page switches and process are unified	1.00	95.000	4.716	1.499	0.154	−8.976	0.000
	2.00	95.000	6.284	0.808	0.083		
B5. The interface of the app allows me to use all the functions it	1.00	95.000	4.526	1.649	0.169	−9.243	0.000
offers (such as input messages, responding to reminders, and reading messages)	2.00	95.000	6.284	0.846	0.087		
B6. I was looking for that the app has all the functionality and	1.00	95.000	4.684	1.401	0.144	−10.786	0.000
processing power	2.00	95.000	6.453	0.769	0.079		
C1. This app is good for my health.	1.00	95.000	4.453	1.556	0.160	−10.284	0.000
	2.00	95.000	6.326	0.856	0.088		
C2. The app has improved my access to healthcare	1.00	95.000	4.632	1.474	0.151	−10.422	0.000
	2.00	95.000	6.421	0.793	0.081		
C3. The app helps me manage my health more effectively.	1.00	95.000	4.484	1.501	0.154	−11.705	0.000
	2.00	95.000	6.495	0.742	0.076		
C4. This app makes it easy for me to communicate with my	1.00	95.000	4.558	1.838	0.189	−9.842	0.000
healthcare staff	2.00	95.000	6.537	0.681	0.070		
C5. Using the app gave me more opportunities to interact with my	1.00	95.000	4.463	1.398	0.143	−12.911	0.000
healthcare staff	2.00	95.000	6.558	0.740	0.076		
C6. I trust my healthcare staff to receive any messages I send	1.00	95.000	4.484	1.556	0.160	−10.957	0.000
using the app	2.00	95.000	6.442	0.782	0.080		
C7. I feel comfortable using the app to communicate with my	1.00	95.000	4.516	1.458	0.150	−10.850	0.000
healthcare staff	2.00	95.000	6.389	0.842	0.086		

The Pearson’s correlation coefficient was used to assess the correlation among 21 items. As shown in Table 3, all the items were significant, and a positive correlation was found among the values of assessment phase. The correlation coefficient between the score of each item and the total score of the questionnaire was calculated, 0.480∼0.681 (*P* < 0.001), thus, all items were retained.

**TABLE 3:** The Pearson’s correlation coefficient between each item and the total score of the questionnaire.

Item	Overall score
The total score of the questionnaire	1
The app is easy to use (Fairman et al., 2013)	0.580***
It is easy for me to learn to use the app (Parmanto et al., 2013)	0.654***
I like the interface of the app (Pfammatter et al., 2016)	0.643***
The information in this app is very organized, so I can easily find the information I need (Seto et al., 2012)	0.633***
I feel comfortable using this app in various public places (Association of American Medical Colleges [AAMC], 2021)	0.659***
Using this app doesn’t take much of my time (National Health Service [NHS], 2019)	0.681***
I will use the app again (Shah et al., 2021)	0.552***
Overall, I am satisfied with the app (Mann et al., 2020)	0.612***
Whenever I have a wrong operation while using this app, I can easily and quickly correct it (Schinkothe et al., 2020)	0.551***
This mHealth app provides healthcare services in a way that is easy for users to accept (Maramba et al., 2019)	0.480***
The app gives enough feedback and information to let me know where my steps are (Vera et al., 2019)	0.581***
The way and process of page switching are unified (World Health Organization [WHO], 2020)	0.578***
The interface of the app allows me to use all the functions it offers (such as entering messages, responding to alerts, and reading messages) (International Organization for Standardization [ISO], 2018)	0.594***
The app has all the functionality and processing power I expected (Stinson et al., 2006)	0.619***
The app is good for my health (Van Velsen et al., 2018)	0.601***
The app has improved my access to healthcare (Walsh et al., 2017)	0.578***
The app helps me manage my health more effectively (Broekhuis et al., 2021)	0.648***
The app makes it easy for me to communicate with my healthcare staff (Ivory and Marti, 2001)	0.580***
Using this app gives me more opportunities to interact with my medical staff (Peute et al., 2008)	0.622***
I trust my healthcare staff to receive any messages I send using the app (Sevilla-Gonzalez et al., 2020)	0.642***
I feel comfortable using the app to communicate with my healthcare staff (Chin et al., 1988)	0.598***

***P < 0.001.

### Validity Testing of the Questionnaire

#### Content Validity

As shown in Table 4, the CVI of each item in the questionnaire was > 0.790, indicated that the items were comprehensible to the target users (Zamanzadeh et al., 2015). and the CVI of the total score of the questionnaire was 0.952.

**TABLE 4:** Content validity of the questionnaire.

Item	I-CVI	S-CVI
A1. The app is easy to use	1.000	0.952
A3. I like the interface of the app	0.857	
A4. The information in this app is very organized and I can easily find the information I need	1.000	
A5. I feel comfortable using the app in all kinds of public places	0.857	
A6. Using this app doesn’t take much of my time	0.857	
A7. I will use the app again	0.857	
A8. Overall, I’m happy with the app	1.000	
B1. Whenever I have a wrong operation while using the app, I can easily and quickly correct it	1.000	
B2. This is a mHealth app that provides healthcare in a way that users can easily accept	1.000	
B3. The app gives me enough feedback and information to let me know where my steps are going	1.000	
B4. The way and process of page switching are unified	1.000	
B5. The interface of the app allows me to use all the functions it offers (such as entering messages, responding to alerts, and reading messages)	1.000	
B6. I was looking for that the app has all the functionality and processing power	1.000	
C1. This app is good for my health	1.000	
C2. The app has improved my access to healthcare	1.000	
C3. The app helps me manage my health more effectively	1.000	
C4. This app makes it easy for me to communicate with my healthcare staff	1.000	
C5. Using the app gave me more opportunities to interact with my healthcare staff	1.000	
C6. I trust my healthcare staff to receive any messages by the app	0.857	
C7. I feel comfortable using the app to communicate with my healthcare staff	0.857	

#### Correlation Analysis

Correlation analysis was used to study the correlation between the overall usability and satisfaction of the questionnaire, the arrangement of system information, and the efficiency of the questionnaire. The Pearson’s correlation coefficient was used to indicate the strength of the correlation. The results of correlation analysis revealed that, usability and satisfaction, arrangement of system information, and efficiency of the questionnaire were all significant, and the correlation coefficients between each dimension and the total score were 0.797, 0.711, and 0.760 respectively, indicating the existence of positive correlations among the usability and satisfaction, the arrangement of system information, and the efficiency of the questionnaire (Table 5).

**TABLE 5:** The correlation analysis of three dimensions using the Pearson’s correlation coefficient.

	Total score of the questionnaire	Ease of use and satisfaction	Arrangement of system information	Efficiency
Total score of the questionnaire	1			
Ease of use and satisfaction	0.797***	1		
Arrangement of system information	0.711***	0.370***	1	
Efficiency	0.760***	0.371***	0.342***	1

***P < 0.001.

### Structural Validity

#### Exploratory Factor Analysis

The validity of the questionnaire was assessed through EFA of the values of Kaiser-Meyer-Olkin (KMO), common degree (common factor variance), variance interpretation rate, load factor, and other indicators. The corresponding common degree values of the research items were all higher than 0.400, Kaiser gave the commonly used KMO measurement standard: above 0.9 means it is very suitable; 0.8 means suitable; 0.7 means general; 0.6 or below means not suitable. The KMO value of this study was 0.932 > 0.800, suitable for factor analysis which indicated that the questionnaire had an acceptable validity. The variance interpretation rates of the three factors were 23.65%, 21.76%, and 18.42%, respectively, and the cumulative variance interpretation rate after rotation was 63.830% > 60%, which revealed that all the information could be extracted effectively. After rotating the component matrix for each question in the questionnaire, the load factor of each item in a certain dimension was > 0.500. Therefore, the validity of the questionnaire was noticeable and the questionnaire was efficient (Table 6).

**TABLE 6:** The results of EFA.

Item	Load factor			Common degree (common factor variance)
	Load factor #1	Load factor #2	Load factor #3	
A1 The app is easy to use	0.751	0.105	0.092	0.583
A2 It is easy for me to learn to use the app	0.735	0.195	0.163	0.605
A3 I like the interface of the app	0.760	0.177	0.129	0.626
A4 I need that The information in the app is very organized and I can easily find the information	0.699	0.177	0.183	0.553
A5 I feel comfortable using the app in all kinds of public places	0.725	0.163	0.220	0.601
A6 Using this app doesn’t take much of my time	0.750	0.220	0.169	0.640
A7 I will use the app again	0.889	0.003	−0.015	0.790
A8 Overall, I’m happy with the app	0.772	0.092	0.146	0.626
B1 Whenever I make an error while using the app, I can easily and quickly correct it	0.176	0.077	0.768	0.627
B2 The mHealth app offers healthcare in a way that is easy for users to accept	0.002	0.033	0.881	0.777
B3 The app gives me enough feedback and information to let me know where my steps are going	0.179	0.139	0.749	0.613
B4 The way and process of page switching are unified	0.152	0.179	0.732	0.591
B5 The interface of the app allows me to use all the functions it offers (such as entering messages, responding to alerts, and reading messages)	0.153	0.173	0.766	0.641
B6 I was looking for that the app had all the functionality and processing power	0.214	0.198	0.721	0.605
C1 The app is good for my health	0.152	0.734	0.141	0.581
C2 The app has improved my access to healthcare	0.117	0.752	0.124	0.595
C3 The app helps me manage my health more effectively	0.185	0.773	0.156	0.657
C4 The app makes it easy for me to communicate with my healthcare staff	0.026	0.900	0.063	0.815
C5 Using the app gave me more opportunities to interact with my healthcare staff	0.175	0.747	0.144	0.609
C6 Using the app that I trust my healthcare staff to receive any messages	0.211	0.752	0.137	0.629
C7 I feel comfortable using the app to communicate with my healthcare staff	0.134	0.782	0.102	0.641
Characteristic root value (before rotation)	7.714	3.025	2.665	–
Variance interpretation rate % (before rotation)	36.73%	14.41%	12.69%	–
Accumulated variance interpretation rate% (before rotation)	36.73%	51.14%	63.83%	–
The characteristic root (after rotation)	4.967	4.571	3.867	–
Variance interpretation rate % (after rotation)	23.65%	21.76%	18.42%	–
Accumulated variance interpretation rate % (after rotation)	23.65%	45.42%	63.83%	–
KMO value	0.932			–
Barthes spherical value	4118.882			–
df	210			–
*P*-value	0			–

#### Confirmatory Factor Analysis

**FIGURE 1:**
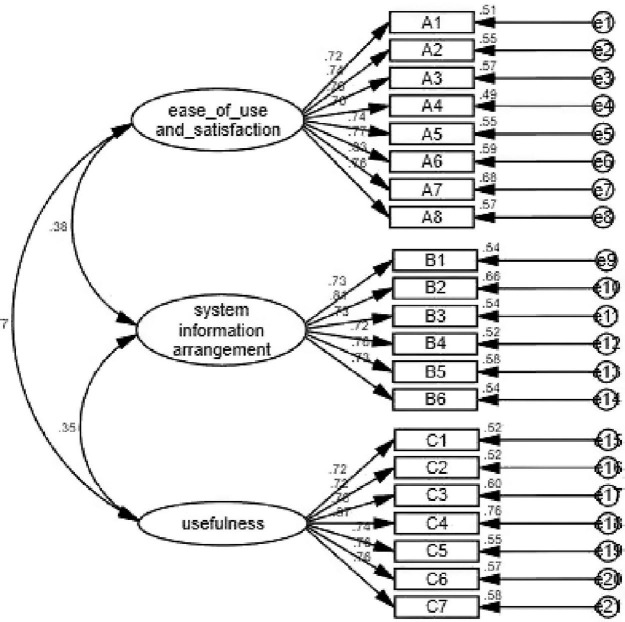
The results of CFA.

Confirmatory factor analysis was used in this study to indicate whether the structure of the questionnaire was consistent with the theoretical structure of the original questionnaire. As shown in Figure 1, when the measurement model was established, the maximum likelihood estimation method was used to estimate the parameters of the model by maximizing the likelihood function. The estimated parameters were load factor, equation error, and error covariance. The most direct parameter that was used to evaluate the goodness of fit of the model. The evaluation of goodness-of-fit the model included the testing of degree of fitting and testing of parameters. In this study, the minimum discrepancy/degrees of freedom (CMIN/DF), normed fit index (NFI), incremental fit index (IFI), Tucker-Lewis index (TLI), comparative fit index (CFI), goodness-of-fit index (GFI), adjusted GFI (AGFI), root mean square error of approximation (RMSEA), and other model fitness indices all met the requirements, indicating that the fitness of the model was good (Table 7).

**TABLE 7:** The evaluation of the goodness-of-fit of the model.

CMIN	Df	CMIN/DF	NFI	IFI	TLI	CFI	GFI	AGFI	RMSEA
338.494	186	1.820	0.920	0.962	0.957	0.962	0.922	0.904	0.049
Suggested value		< 3	> 0.9	>0.9	> 0.8	>0.9	> 0.9	>0.9	< 0.080

#### Analysis of Convergent Validity and Discriminant Validity

In this study, the composite reliability (CR) and average variance extracted (AVE) values were used as the evaluation criteria for convergence validity. When the CR value of each factor is > 0.700 and the AVE value is > 0.500, it is generally considered that the convergent validity is good. In addition, when the square root value of AVE of each factor is higher than the correlation coefficient between this factor and other factors, it indicates a high discriminant validity. The results of convergence validity and discriminant validity are presented in Table 8. As shown in Table 8, the mean AVE value of each dimension is greater than 0.5, and the mean CR value is greater than 0.700, indicating that the convergence validity of the questionnaire is noticeable.

**TABLE 8:** The results of the convergent validity analysis.

Relationship between variables			Estimated coefficient	Standard error	Critical value	*P*	Load factor	AVE	CR
A1	< —	Ease of use and satisfaction	1.000				0.716	0.565	0.912
A2	< —	Ease of use and satisfaction	1.030	0.078	13.251	***	0.743		
A3	< —	Ease of use and satisfaction	1.081	0.080	13.506	***	0.757		
A4	< —	Ease of use and satisfaction	0.972	0.078	12.478	***	0.700		
A5	< —	Ease of use and satisfaction	0.984	0.074	13.219	***	0.741		
A6	< —	Ease of use and satisfaction	1.056	0.077	13.695	***	0.768		
A7	< —	Ease of use and satisfaction	1.207	0.082	14.740	***	0.827		
A8	< —	Ease of use and satisfaction	1.048	0.078	13.466	***	0.755		
B1	< —	Arrangement of system information	1.000				0.735	0.562	0.885
B2	< —	Arrangement of system information	1.220	0.084	14.544	***	0.814		
B3	< —	Arrangement of system information	1.044	0.080	13.118	***	0.734		
B4	< —	Arrangement of system information	0.985	0.077	12.838	***	0.719		
B5	< —	Arrangement of system information	1.117	0.082	13.601	***	0.761		
B6	< —	Arrangement of system information	1.006	0.077	13.071	***	0.732		
C1	< —	Efficiency	1.000				0.723	0.586	0.908
C2	< —	Efficiency	0.941	0.073	12.971	***	0.720		
C3	< —	Efficiency	1.047	0.075	13.983	***	0.775		
C4	< —	Efficiency	1.289	0.082	15.734	***	0.873		
C5	< —	Efficiency	1.002	0.075	13.353	***	0.741		
C6	< —	Efficiency	1.032	0.076	13.620	***	0.755		
C7	< —	Efficiency	1.023	0.075	13.697	***	0.759		

***P < 0.001.

According to the results of the discriminant validity analysis (Table 9), the square root of the AVE was greater than the value of the correlation with other factors, thus, the discriminant validity (among internal factors) of each variable was very promising.

**TABLE 9:** The results of the discriminant validity analysis using the Pearson’s correlation coefficient.

	Ease of use and satisfaction	Arrangement of system information	Efficiency
Ease of use and satisfaction	0.752		
Arrangement of system information	0.370**	0.750	
Efficiency	0.371**	0.342**	0.765

**P < 0.01.

#### Reliability Testing of the Questionnaire

The Cronbach’s α coefficient of the Chinese version of the MAUQ was 0.912, higher α value suggests greater internal reliability and more than0.700 is acceptable as good internal reliability (Allen et al., 2014). The values of test-retest reliability were 0.869, and the values of split-half reliability were 0.701. The reliability of each dimension of the questionnaire is presented in Table 10.

**TABLE 10:** Reliability of each dimension of the questionnaire.

Dimensionality	Cronbach’s α coefficient	Test-retest reliability	Split-half reliability
Ease of use and satisfaction	0.912	0.840	0.916
Arrangement of system information	0.884	0.853	0.888
Efficiency	0.907	0.809	0.908
Overall	0.912	0.869	0.701

## Discussion

This study described the process of cross-cultural translation and adaption of the MAUQ questionnaire Semantic and cultural equivalence was achieved between the two versions and the adapted one showed excellent internal consistency and good validity finally.

The development of new questionnaires requires the joint efforts of members of professional research teams, while attaining the objective is costly and time-consuming. Therefore, it is recommended to adapt the established, reliable, and available questionnaires and record their validity in the language used (Mohamad Marzuki et al., 2018). However, due to the linguistic and cultural differences between Chinese and English, literal translation may cause ambiguity in the meaning expressed in a part of the content, hindering the production of an authenticated questionnaire. Therefore, cross-cultural adaptation of the questionnaire is necessary (Epstein et al., 2015). The present study used the Brislin’s translation model (Brislin, 1976) for translation, and the translation results showed that the majority of elements were easy to understand, while few items were adjusted after translation according to Chinese expression habits.

The verification analysis of the questionnaire is an important step to ensure that the composition of the translated version of the questionnaire is as same as that of the original version. CVI and reliability index were used in the current research. The CVI is basically used to measure the validity of the questionnaire (Maramba et al., 2019). It is easy to measure and understand, and the content of each item can be modified or deleted according to the detailed information of each item (Polit et al., 2007; Zamanzadeh et al., 2015). The results of this study showed that the CVI value of each item was 0.857∼1.000, and the total CVI was 0.952 > 0.790 (benchmark value), indicating that the Chinese version of the MAUQ could properly represent each item. Reliability is the degree to which an assessment tool produces stable and consistent results. In the present study, the Cronbach’s α coefficient, split-half reliability, and test-retest reliability were used to evaluate the reliability of the questionnaire. The results showed that the values of Cronbach’s α coefficient, test-retest reliability, and split-half reliability were 0.912 > 0.800, 0.869 > 0.800, and 0.701 > 0.700, respectively. The results confirmed that the questionnaire had a good internal consistency and stability.

In the present study, the reliability and validity of the MAUQ were tested to provide an evaluation tool for mobile medical services in China. However, there are some limitations in this study. Firstly, 346 patients recruited in this study were outpatients from a third-class hospital, particularly youth who were familiar with smart phones. The results of this study have not been generalized to a wider population, especially the elderly. Secondly, this study only concentrated on the evaluation of the patient version of the “Good Doctor” app, thus, the scope of our research was limited, and it could not be applied to other versions (provider version or medical version) of different mHealth apps. In the future research, we will concentrate on the usability testing tools for mHealth apps in all aspects, so as to further promote the development of mHealth apps.

## Conclusion

The Chinese version of the MAUQ, an interactive mHealth app for patients, contains 21 items and 3 dimensions, which is consistent with the theoretical structure of the original questionnaire and has a high reliability and validity. All indicators of the Chinese version of the MAUQ could meet the requirements of the measurement, which could effectively and scientifically evaluate the patient version of the interactive mHealth app.

## Data Availability Statement

The raw data supporting the conclusions of this article will be made available by the authors, without undue reservation.

## Ethics Statement

The studies involving human participants were reviewed and approved by Qilu Hospital of Shandong University. The patients/participants provided their written informed consent to participate in this study.

## Author Contributions

SZ and YC contributed to the study design, implementation, analysis, and manuscript writing. HC and KL contributed to the statistical design and analysis. XL, JZ, and YL contributed to the analysis of the data. PD contributed to the translation of the scale. All authors contributed to the article and approved the submitted version.

## Conflict of Interest

The authors declare that the research was conducted in the absence of any commercial or financial relationships that could be construed as a potential conflict of interest.

## Publisher’s Note

All claims expressed in this article are solely those of the authors and do not necessarily represent those of their affiliated organizations, or those of the publisher, the editors and the reviewers. Any product that may be evaluated in this article, or claim that may be made by its manufacturer, is not guaranteed or endorsed by the publisher.

## Abbreviations

CVI: content validity index
KMO: Kaiser Meyer Olkin
MAUQ: mHealth app usability questionnaire
mHealth: mobile health.
